# Two cases of nivolumab plus ipilimumab therapy for dialysis patients with advanced bone metastasis from renal cell carcinoma

**DOI:** 10.1007/s13730-022-00753-8

**Published:** 2022-11-19

**Authors:** Mika Takahashi, Minika Takishita, Yukako Yamazato, Hiroaki Kakinoki, Kazuma Udo, Shohei Tobu, Mitsuru Noguchi

**Affiliations:** Department of Urology, Faculty of Medicine, Saga University, 5 Chome-1-1, Nabeshima, Saga-City, Saga 849-8501 Japan

**Keywords:** Bone metastasis, Dialysis, Immune checkpoint inhibitor, Nivolumab plus ipilimumab therapy, Renal cell carcinoma

## Abstract

Nivolumab and ipilimumab are immune checkpoint inhibitors. Combination therapy with these two drugs has been shown to improve the outcome of advanced renal cell carcinoma. However, data about the safety and the efficacy of combination therapy with these two drugs in hemodialysis patients are small. A 59-year-old male hemodialysis patient presented with bone metastasis from renal cell carcinoma, which was located at the right femur. He received nivolumab plus ipilimumab therapy. At 7 months after treatment, he was diagnosed with diabetes as an immune-related adverse event. He was managed with insulin therapy. At 11 months after treatment, CT revealed cytoreduction of metastasis. A 74-year-old male hemodialysis patient presented with bone metastasis of renal cell carcinoma located at the sacrum and left scapula. He received nivolumab plus ipilimumab therapy. At 6 months after treatment, CT showed no progression of metastasis. Nivolumab and ipilimumab therapy might be a viable treatment for hemodialysis patients with bone metastasis from renal cell carcinoma. However, close attention should be paid immune-related adverse events in such patients.

## Introduction

Nivolumab and ipilimumab are immune checkpoint inhibitors (ICIs) that target the programmed death-1 (PD-1) and cytotoxic T-lymphocyte associated protein-4 (CTLA-4) pathways. Combination therapy with these two drugs has been shown to improve the outcome in patients with International Metastatic Renal Cell Carcinoma Database Consortium (IMDC) intermediate risk and poor risk metastatic renal cell carcinoma (mRCC) in comparison with sunitinib treatment [1].

However, data concerning the safety and the efficacy of ICI monotherapy or combination therapy in hemodialysis patients are small, because such patients have been excluded from clinical trials [1, 2]. After radical nephrectomy due to treatment of RCC some patients are diagnosed with CKD, with some receiving hemodialysis. Although several case studies have reported that hemodialysis patients can be treated safely and efficaciously with nivolumab plus ipilimumab combination therapy. We experienced two cases in which hemodialysis patients received nivolumab plus ipilimumab therapy for mRCC.

## Case report

### Case 1

The patient was a 59-year-old man who presented with an incidentally discovered left renal mass 10 years previously. He underwent left radical nephrectomy, and a pathological examination revealed pT1bpN0M0, clear cell renal cell carcinoma. After the operation he was diagnosed with CKD, and had received dialysis for 5 years. At 4 years, after radical nephrectomy, a left lung mass was detected on CT, and was resected by thoracoscopy. The pathological diagnosis was metastasis of clear cell renal cell carcinoma. A CT scan from 3 years previously showed right femur fracture due to bone metastasis. The fracture was surgically treated with intramedullary nailing (Table 1). Unfortunately, metastatic bone lesion had grown bigger, and he was classified as intermediate risk according to the IMDC risk classification. Thus, combination treatment with nivolumab (240 mg/body) plus ipilimumab (1 mg/kg) every 3 weeks for 4 cycles was initiated, followed by nivolumab monotherapy (240 mg/body) every 2 weeks as first-line therapy for mRCC. Treatments were performed on Thursday morning, while dialysis performed on Tuesday, Thursday, and Saturday evenings. ICIs are not removed by hemodialysis [3–6]. Therefore, this patient continued receiving hemodialysis as he had before treatment.

**Table 1:** Clinical characteristic of two patients

Case	Initial Stage Histology	Dialysis period	Previous treatment	Metastasis sites	Medical histories	IMDC classification	irAEs	Cancer outcome
1	pT1bpN0M0 Clear cell renal cell carcinoma	5 years	Lung metastasis operation Right femur metastasis operation	Right femur	IgA nephropathy, Hypertension, CKD Stage 5	Intermediate	Diabetes	Cytoreduction of metastasis lesion
2	pT1bN0M0 Clear cell renal cell carcinoma	9 years	No treatment	Sacrum, Left scapula	Diabetes, Hypothyroidism, Hyperuricemia, Sleep apnea syndrome, CKD Stage 5	Intermediate	None	No change, either

**Fig. 1:**
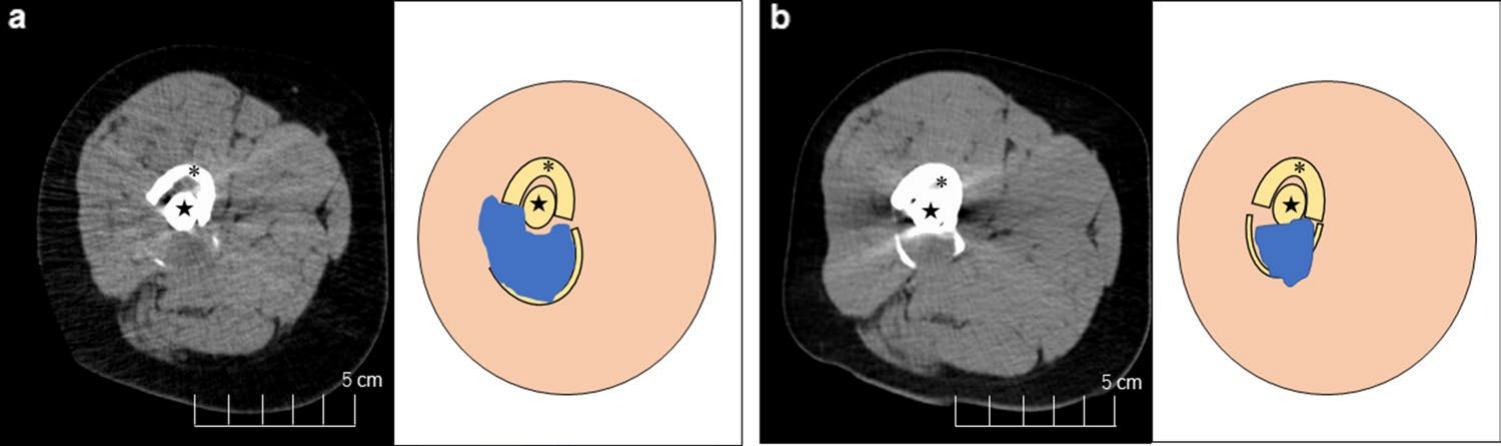
Case 1 **a** Before therapy. **b** Eleven months after the initiation of therapy. The left figures show the CT findings at the right femur metastasis, and the right figures show schematic diagrams of the left CT scan. Tumor reduction rate is 23%. * and ★ indicate the right femur and intramedullary nail, respectively. The blue and beige areas in the right figures indicate bone metastasis of the right femur and right thighs, respectively

At 7 months after the start of treatment, hyperglycemia was detected. Thus, he was diagnosed with diabetes as an immune-related adverse event (irAE). Intensive-insulin therapy was performed and he continued to receive insulin therapy after the treatment. At 11 months after the start of treatment, CT revealed cytoreduction of the metastatic lesion (Fig. 1). He did not experience any other irAEs (e.g., hepatitis, pneumonitis, endocrine irAEs, cutaneous irAEs, or gastrointestinal diarrhea).

### Case 2

The patient was a 74-year-old man who presented with an incidentally discovered right renal mass 19 years previously. He underwent right radical nephrectomy, and a pathological examination revealed pT1bN0M0, clear cell renal cell carcinoma. After the operation he was diagnosed with CKD, and had been receiving dialysis for 9 years. CT revealed metastasis to the sacrum and left scapula (Table 1). He was classified as intermediate risk according to the IMDC risk classification. Thus, combination treatment with nivolumab (240 mg/body) plus ipilimumab (1 mg/kg) every 3 weeks for 4 cycles, followed by nivolumab monotherapy (240 mg/body) every 2 weeks was initiated as first-line therapy for mRCC. Therapy was administered on Thursday morning, while dialysis was performed on Monday, Wednesday and Friday evenings. ICIs are not removed by hemodialysis [3–6]. Therefore, this patient also continued receiving hemodialysis as he had before treatment.

**Fig. 2:**
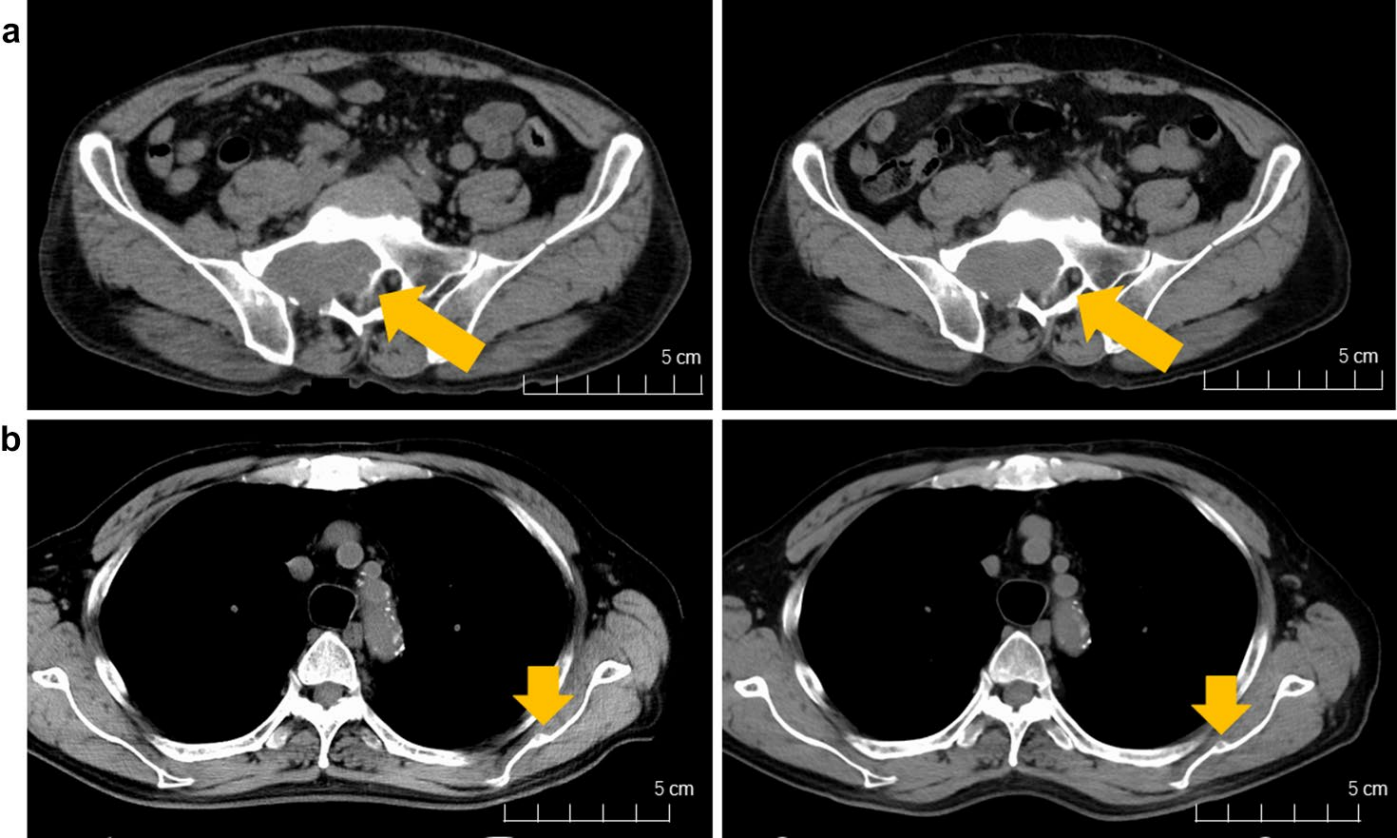
Case 2 **a** Sacrum metastasis before therapy (left) and after 6 months of therapy (right). **b** Left scapula metastasis before therapy (left) and after 6 months of therapy (right). The yellow arrows indicate the location of the sacrum and left scapula metastasis

At 6 months after the start of treatment, CT showed no marked change in the size of the metastatic lesions of the sacrum and left scapula (Fig. 2). ICI therapy was continued without the development of irAEs, including hepatitis, pneumonitis, endocrine irAEs, cutaneous irAEs, gastrointestinal diarrhea, and diabetes.

## Discussion

The renal function has no effect on the pharmacokinetic activity of monoclonal antibodies, such as nivolumab and ipilimumab [3, 4], and no significant effect on the clearance of the drugs, because amino acids are as endogenous as immunoglobulin G (IgG) [3, 5, 6]. Consequently, nivolumab and ipilimumab do not require dose adjustment for patients with renal dysfunction [5, 6]. Furthermore, these drugs are not removed by hemodialysis because of their large molecular weight (145,000 and 148,000, respectively) [5, 6]. Case 1 had been using APS^®^-25SA dialyzer, while Case 2 had been using PES-21Gα eco dialyzer. Therefore, we administered ipilimumab and nivolumab at the conventional doses and for the conventional duration of administration. Furthermore, we did not need to arrange the day of administration or the day on which they performed dialysis.

While patients receiving dialysis have impaired immune systems, showing a reduced immune cell activity and low antibody levels [7], dialysis patients in some case reports have responded to ICIs as well as non-dialysis patients. Sester et al. reported that the maturation of helper T cells in dialysis patients is sustained, these patients still presented with significantly elevated Th1 levels, leading to an increased Th1/Th2 ratio [8].

Ipilimumab is a fully human IgG-1 antibody that binds to the CTLA-4 molecule, a coinhibitory immune checkpoint expressed on activated *T* cells. Blocking signaling through the CTLA-4 molecule results in unrestrained activation of T cells [9, 10], which can have an anticancer effect. On the other hand, nivolumab is a fully human IgG-4 PD-1 ICI antibody that selectively blocks the interaction between PD-1, which is expressed on activated *T* cells, and its ligands PD-L1 and PD-L2, which are expressed on immune cells and tumor cells. As such, it suppresses cancer cell growth. ICIs regulate pathways of *T* cells to enhance the anti-tumor immune response [1]. Therefore, we suspect that because the Th1 level in dialysis patients is relatively high, ICIs more readily responded to tumor cells, despite the relatively reduced immune cell activity and low antibody levels in these patients. Furthermore, the dialysis period, medical history, age, and other factors might also influence the patient response to ICI therapy.

Several case studies have reported on the cancer outcomes of dialysis patients using ICIs (atezolizumab, nivolumab, pembrolizumab, and ipilimumab plus nivolumab) and demonstrated that the complete remission (CR) rate was 28%, the partial remission (PR) rate was 4%, the stable disease (SD) rate was 30% and the progressive disease (PD) rate was 38% [11]. On the other hand, there is a report that over 80% of patients had either the partial or complete response to treatment [12]. Our cases showed reduction in the size of the lesions and no change in the size of the lesion.

Next, it should be noted that ICIs were initially approved for melanoma [10]; however, over the years, they can be a very effective in treatments for various other cancers, such as renal cell carcinoma, urothelial carcinoma, non-small-cell lung cancer, and head and neck cancer. A total of 15% of dialysis patients showed severe irAEs (grade 3 and 4), while 34% showed mild irAEs (grade 1 and 2) [11]. Consequently, the incidence of irAEs in patients with end stage kidney disease (ESKD) who were receiving dialysis was similar to that in the general population. It has been reported that hematologic, dermatologic and gastrointestinal toxicities are the most common types of organ toxicity [11]. On the other hand, Jamie et al. reported that 26 ESKD patients with melanoma, RCC, urothelial carcinoma, non-small-cell lung carcinoma, and squamous cell skin carcinoma receiving ICI therapy developed irAEs (15%), except for kidney transplant rejection [12]. Therefore, ESKD patients are not more likely to develop irAEs than non ESKD patients. One of our patients developed diabetes as an irAE.

Severe irAEs including encephalitis, pneumonitis, and myocarditis, occurred at rates similar to the general population [11], with incidence estimates of below 1% [13, 14]. Furthermore, we summarized current reports concerning nivolumab plus ipilimumab therapy for dialysis patients with RCC in Table 2. If patients treated with ICI therapy developed irAE, they were treated with corticosteroids or endocrine replacement therapy, in keeping with American Society of Clinical Oncology and European Society of Medical Oncology guidelines [20, 21]. However, dialysis patients have multiple other diseases, including connective tissue disease, diabetes and heart failure. Therefore, we should be considered corticosteroid dose [11]. Therefore, we should also keep in mind the risk and the benefit of steroids as a treatment for dialysis patients’ irAEs. We should frequently check the laboratory data, oxygen saturation, and chest X-ray findings, as well as other information, to ensure that irAEs are detected early. More data and further analyses will be necessary to better understand the characteristics of ICI therapies and to improve malignancy outcomes in patients receiving dialysis.

**Table 2:** Summary of patient data from seven published cases of nivolumab plus ipilimumab therapy for dialysis patients with RCC

Case	Gender	Age	Dialysis period	Histology	irAEs	Reference No.
1	Male	70	1 month	Clear cell RCC	Adrenal insufficiency (grade 3)	[15]
2	Male	63	25 years	L: Papillary RCC	None	[15]
				R: Clear cell RCC		
3	Male	40	2 years	Clear cell RCC	Adrenal insufficiency (grade 3)	[15]
4	Male	53	6 years	Clear cell RCC with sarcomatoid dedifferentiation	Lung damage (grade 1)	[16]
5	Male	73	21 years	Not performed biopsy	None	[17]
6	Male	77	11 years	Clear cell RCC	None	[18]
7	Male	66	1 year	Papillary RCC and UC	None	[19]

*RCC* renal cell carcinoma, *UC* urotherial carcinoma

On the other hand, the survival of patients with cancer is dependent on the type of cancer. ICI therapy have improved various cancer outcome in comparison with prior therapies. Therefore, we should select the best treatment depending on the type of cancer, their medical histories, and the prognosis. Therefore, close attention should be paid to such patients’ irAEs.

## Conclusion

Although case 1 patient was diagnosed with diabetes as in irAE, two patients also have not been diagnosed with any other irAEs including hepatitis, pneumonitis, endocrine irAEs, cutaneous irAEs, and gastrointestinal diarrhea. Nivolumab and ipilimumab therapy might be a viable treatment in hemodialysis patients with bone metastasis from renal cell carcinoma. Close attention should be paid to such patients’ irAEs.
